# Swelling of the Right Arm During a Nuclear Medicine Therapy for Metastatic Pheochromocytoma

**DOI:** 10.1016/j.aace.2024.11.003

**Published:** 2024-11-18

**Authors:** Run Yu, Linda Gardner, Zachary Ells, Magnus Dahlbom, Ali Salavati, Shadfar Bahri

**Affiliations:** 1 Division of Endocrinology, UCLA David Geffen School of Medicine, Los Angeles, California; 2 Ahmanson Translational Imaging Division, UCLA David Geffen School of Medicine, Los Angeles, California

## Case Presentation

**Fig 1:**
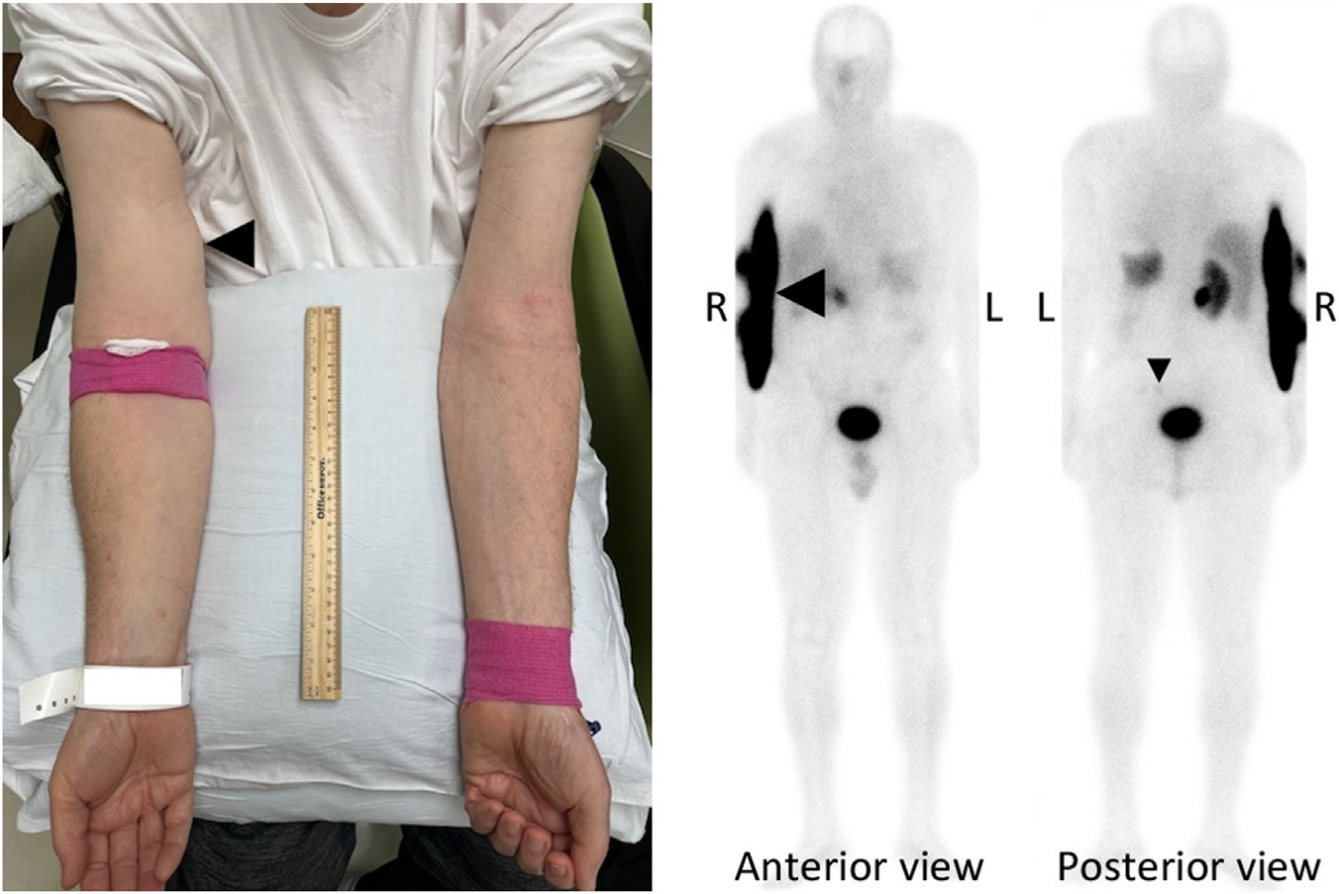

A 77-year-old man with metastatic pheochromocytoma underwent peptide receptor radionuclide therapy (PRRT) with lutetium-177 dotatate as Lutathera (LUT). He had been diagnosed with left adrenal pheochromocytoma and received left adrenalectomy 20 years before. He had developed pheochromocytoma recurrence in the left adrenal bed and metastasis in lymph nodes and skeleton and received surgical resection and radiation 6 years before. The tumor burden had remained stable until 2 years before. He had then received PRRT with 4 doses of LUT. The tumor burden had remained stable for 6 months and then progressed again. Metanephrine was undetectable (normal, <57 pg/mL), and the normetanephrine level was 3322 pg/mL (normal, <148 pg/mL). His blood pressure was well controlled by prazosin 2 mg twice daily and metoprolol 25 mg daily. Salvage PRRT with 2 doses of LUT was started. During the administration of the first salvage LUT dose, 200 mCi of LUT in 25 mL was piggybacked over 80 minutes on an intravenous line with ongoing amino acids infusion inserted at the right antecubital fossa. At the end of LUT infusion, staff noted right arm swelling (Fig. 1, left) and immediately transferred the amino acid line to the left arm.

## What is the diagnosis?

### Answer

**Fig 2:**
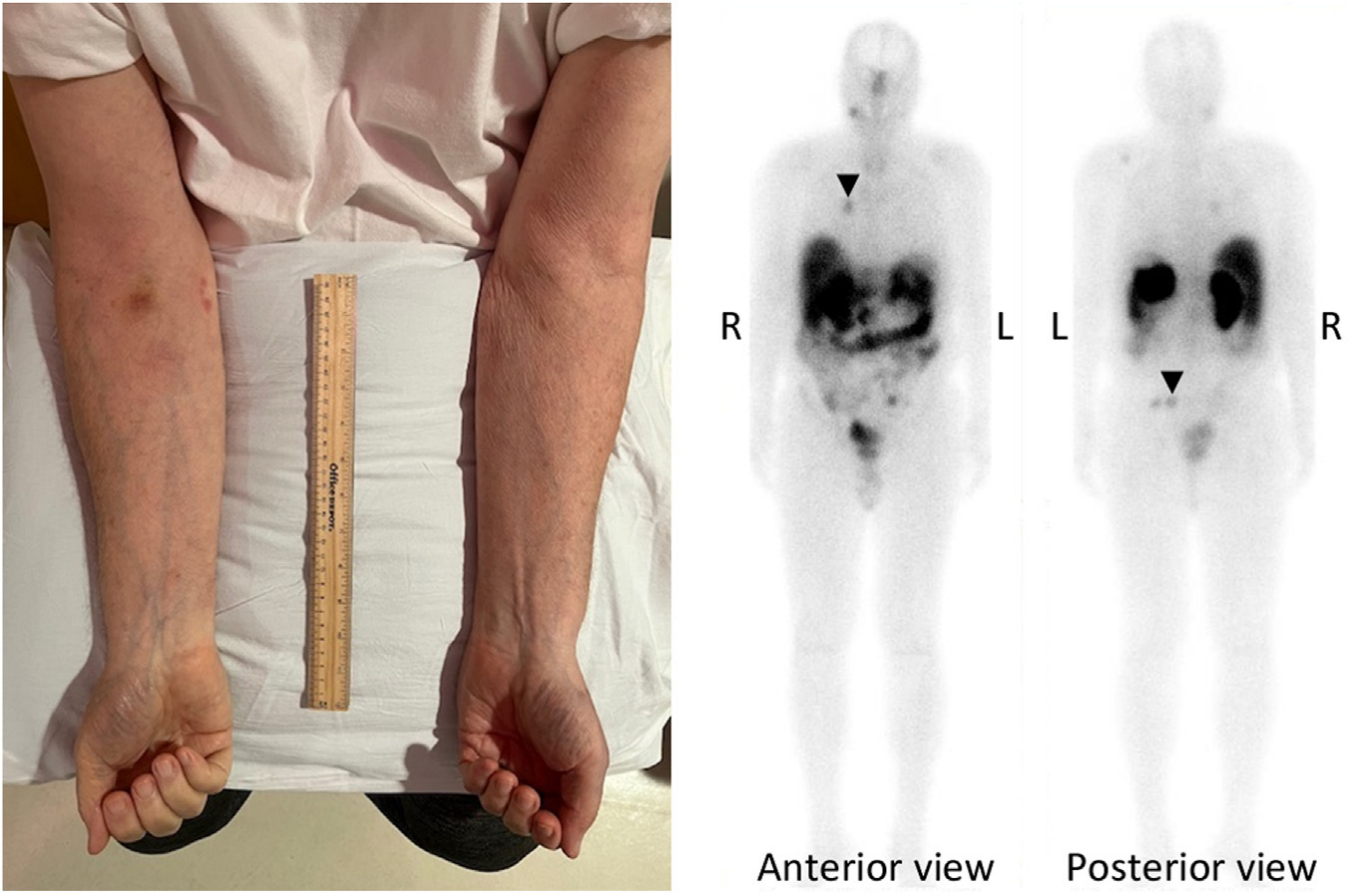

LUT extravasation in a patient with metastatic pheochromocytoma. Four hours after LUT infusion, whole-body planar imaging with single photon emission computed tomography showed large accumulation of radioactivity in the right arm with minimal radioactivity at the metastatic lesions (Fig. 1, right), consistent with LUT extravasation. Salvage PRRT with LUT is safe and efficacious for patients with metastatic neuroendocrine carcinomas, including metastatic pheochromocytoma, who have responded to the initial 4 doses of LUT.^1^ As this patient with progressive metastatic pheochromocytoma had satisfactory response to his initial PRRT, salvage PRRT is appropriate and indicated. LUT extravasation is a rare complication of PRRT.^2^^,^^3^ Although sounding catastrophic, LUT extravasation usually does not cause significant clinical consequences.^2^^,^^3^ As the extravasated LUT is drained out of the local tissue around infusion site in hours, no local tissue damage due to beta radiation has been reported, and the target tumor lesions do receive the appropriate doses of LUT.^2^^,^^3^ The extravasation site was treated with warm compression, and the right arm was raised above the heart to facilitate LUT drainage. Forty-eight hours after the LUT extravasation, the right arm swelling was resolved, LUT extravasation was completely cleared, and the target lesions received the intended LUT doses (Fig. 2).

## Disclosure

The authors have no conflicts of interest to disclose.
